# Supplementary material for: Cohort Profile: Virus Watch—understanding community incidence, symptom profiles and transmission of COVID-19 in relation to population movement and behaviour
Source: Int J Epidemiol. 2023 Jun 22;52(5):e263–72. doi: 10.1093/ije/dyad087 (PMC10555858; doi:10.1093/ije/dyad087)
Supplement: dyad087_Supplementary_Data [file dyad087_supplementary_data.docx]

# Supplementary Materials

**Table S1:** Household size in Virus Watch compared to the general population of England

|  | Virus Watch participants | | General population of England (ONS)* |
| --- | --- | --- | --- |
| Number of individuals in household | Frequency | Percentage (%) | Percentage (%) |
| 1 | 9,427 | 33.1 | 28.7 |
| 2 | 12,803 | 44.9 | 34.3 |
| 3 | 2,840 | 10.0 | 16 |
| 4 | 2,463 | 8.6 | 14.5 |
| 5 | 741 | 2.6 | 1.9 |
| 6 | 253 | 0.9 | 0.5 |

*Estimates of household size in England 2021, taken from Office for National Statistics (ONS) 2022 <https://www.ons.gov.uk/peoplepopulationandcommunity/birthsdeathsandmarriages/families/datasets/householdsbyhouseholdsizeregionsofenglandandukconstituentcountries> (1)

**Table S2:** Recruitment rates by method of recruitment into Virus Watch

| Recruitment Method | Number Recruited | Denominator | Recruitment rate |
| --- | --- | --- | --- |
| Facebook campaign 1&2 (30/07/2020 - 26/10/2021) | 17,156 (29.4%) | 633,844 | 2.71% |
| Facebook campaign 3 (27/10/2021 onwards) | 4338 (7.4%) | 284,729 | 1.52% |
| SMS message from GP | 11,180 (19%) | 799,312 | 1.40% |
| Email / friend family | 7561 (12.9%) | 18,255 | 41.46% |
| Other | 4,986 (8.5%) |  |  |
| Postcard/Flyer drop | 3,913 (6.7%) | 200,000 | 1.96% |
| GP letter targeting minority ethnic population | 3,785 (6.5%) | 91,310 | 4.15% |
| Postal recruitment | 3,289 (5.6%) | 49,120 | 6.70% |
| Twitter | 872 (1.5%) |  |  |
| Clinical patient group | 510 (0.9%) |  |  |
| Newspaper/TV | 423 (0.7%) |  |  |
| Whatsapp | 391 (0.7%) |  |  |
| Unknown | 225 (0.4%) |  |  |
| Total | 58,628 |  |  |

**Table S3:** Number of follow-up samples collected between 24/02/2021 and 21/03/2022 from Virus Watch participants enrolled in the vaccination subcohort

| Total number of samples provided | Individuals |
| --- | --- |
| 1 | 3089 |
| 2 | 4146 |
| 3 | 1608 |
| 4 | 912 |
| 5 | 605 |
| 6 | 915 |
| 7 | 1330 |
| 8 | 958 |
| 9 | 892 |
| 10 | 1936 |
| 11 | 3025 |
| 12 or more | 140 |


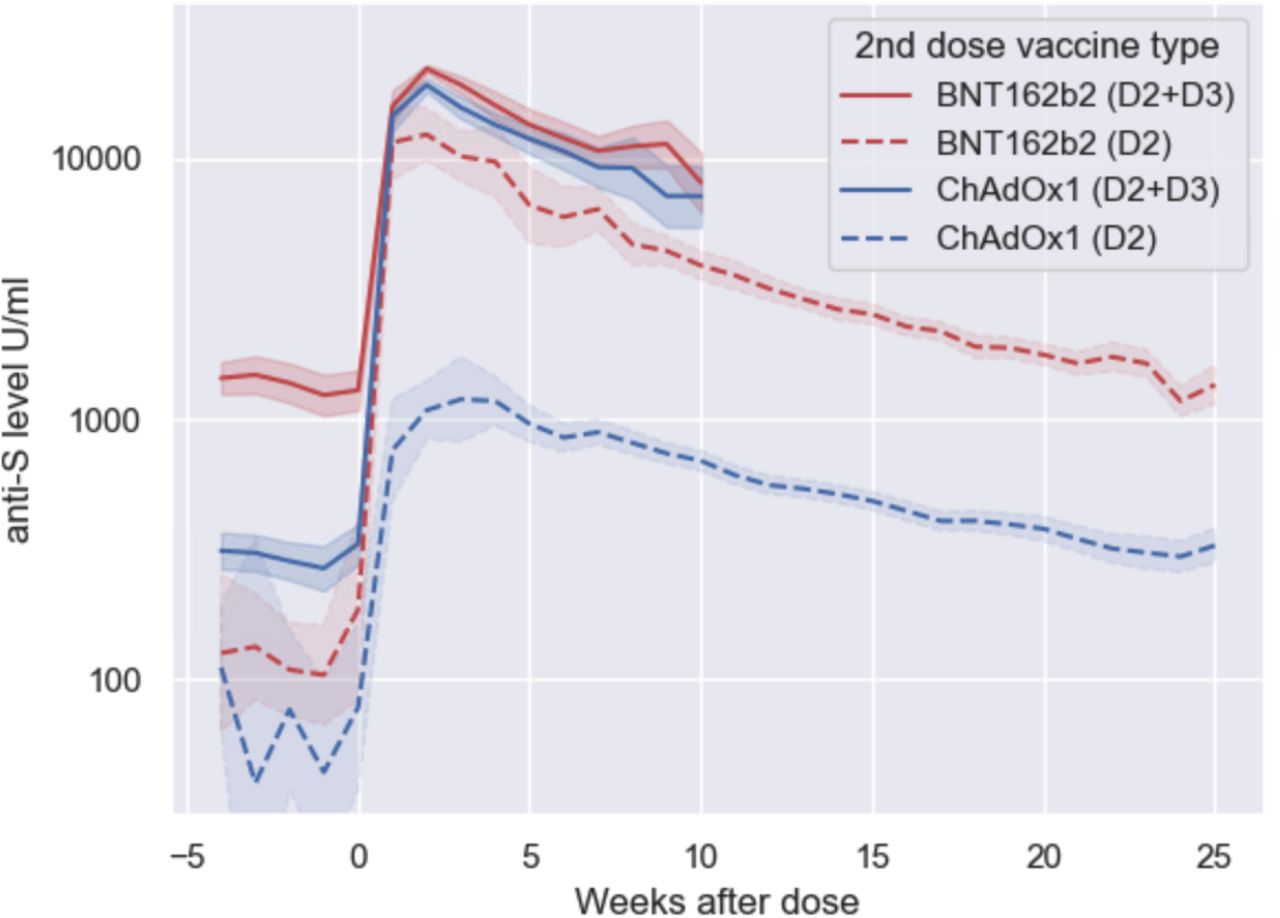


**Figure S1:** Anti-S levels (U/mL) over time since BNT162b2 booster dose (D2+D3) and second vaccine dose (D2) amongst individuals without evidence of prior infection by primary course type. Taken from Yalinsky et al, 2022 (2)

# References

1. Households by household size, regions of England and GB constituent countries - Office for National Statistics. Available from: https://www.ons.gov.uk/peoplepopulationandcommunity/birthsdeathsandmarriages/families/datasets/householdsbyhouseholdsizeregionsofenglandandukconstituentcountries

2. Yavlinsky A, Beale S, Nguyen V, Shrotri M, Byrne T, Geismar C, et al. Anti-spike antibody trajectories in individuals previously immunised with BNT162b2 or ChAdOx1 following a BNT162b2 booster dose. Wellcome Open Res. 2022 Jul 7;7:181.
